# Stage IV anal canal squamous cell carcinoma with long-term survival: a case report

**DOI:** 10.1186/s40792-022-01474-x

**Published:** 2022-06-20

**Authors:** Katsuji Sawai, Takanori Goi, Noriyuki Tagai, Hidetaka Kurebayashi, Mitsuhiro Morikawa, Kenji Koneri, Masato Tamaki, Makoto Murakami, Yasuo Hirono, Hiroyuki Maeda

**Affiliations:** 1 First Department of Surgery, University of Fukui, 23-3 Matsuoka Shimoaizuki Eiheiji-cho, Yoshidagun, Fukui 910-1193 Japan; 2 Cancer Care Promotion Center, Faculty of Medical Sciences, University of Fukui, 23-3 Matsuoka Shimoaizuki Eiheiji-cho, Yoshidagun, Fukui 910-1193 Japan

**Keywords:** Anal squamous cell carcinoma, Multidisciplinary treatment, Long-term survival

## Abstract

**Background:**

Currently, no established standard treatment exists for metastatic anal squamous cell carcinoma. We report a case of complete response in a patient with stage IV anal squamous cell carcinoma after undergoing multidisciplinary treatment.

**Case presentation:**

A 62-year-old woman visited a nearby doctor with a chief concern of severe pain associated with a firm mass in the anus. The patient was diagnosed with anal canal squamous cell carcinoma and liver metastases and referred to First Department of Surgery Faculty of Medicine University of Fukui for treatment. The patient received a TNM classification of T4N0M1 and stage IV. Rectal amputation was performed; however, postoperative complications hindered immediate anticancer therapy and the liver metastases exacerbated. Radiofrequency hyperthermia and systemic chemotherapy were performed 3 months postoperatively. A prominent reduction in the liver metastasis was observed. Lung metastases appeared during the course of systemic chemotherapy. Radiotherapy was performed to treat the lung lesion and resolved. Radiotherapy was also performed for liver metastasis. The lesion in the liver showed resolution after 54 months postoperatively, and treatment with the anticancer drug was discontinued. Ten-year follow-up findings suggested complete resolution of the lesion in response to the treatment protocol followed in this case. This long-term survival was achieved through a multidisciplinary treatment.

**Conclusions:**

The present case suggests that multidisciplinary treatment approach is effective for resolving stage IV anal squamous cell carcinoma, and addition of new anticancer drug therapy may improve the overall prognosis of squamous cell carcinoma.

## Background

Squamous cell carcinoma of the anal canal is a relatively rare malignancy, and 10–20% of the patients are reported to experience metastasis [1]. Combined chemoradiotherapy (CRT) is the standard treatment for locally confined anal squamous cell carcinoma. Large randomized trials have shown that CRT results in good locoregional control and colostomy-free survival rates for patients with locally advanced tumors or carcinomas with lymph node involvement [2, 3]. However, the standard treatment for metastatic anal squamous cell carcinoma has not been established yet. Moreover, patients with localized anal squamous cell carcinoma are expected to have a 5-year overall survival (OS) rate of 78%, but it drops to 19% in patients with distant metastasis [4, 5]. Herein, we report the case of a patient with stage IV anal squamous cell carcinoma who achieved complete resolution after multidisciplinary treatment.

## Case presentation

**Fig. 1:**
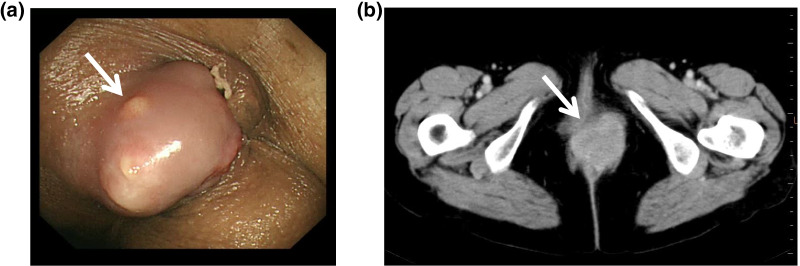
**a** Endoscopic evaluation revealed an anorectal mass (white arrow). **b** An enhanced pelvic CT scan showing the mass at the anal canal (white arrow). *CT* computed tomography

**Fig. 2:**
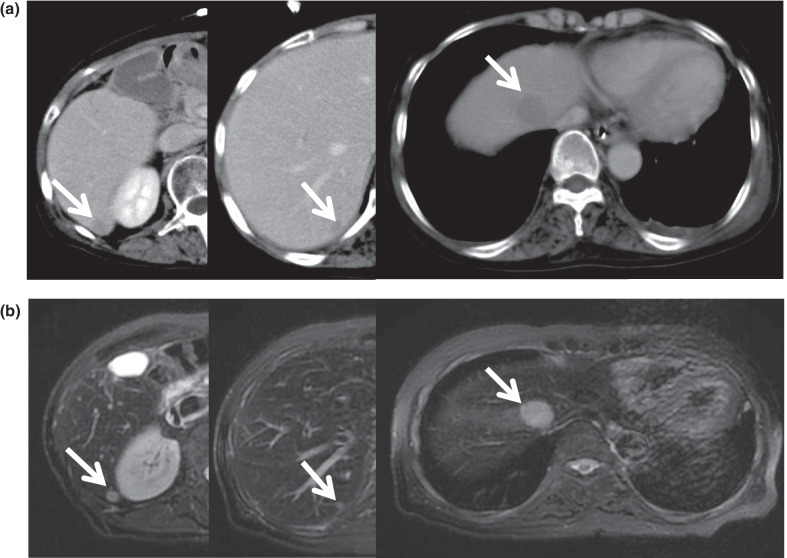
**a**, **b** Preoperative CT scan and MRI of the abdomen showing multiple liver metastases (white arrows). *CT* computed tomography, *MRI* magnetic resonance imaging

A 62-year-old woman was admitted to First Department of Surgery Faculty of Medicine University of Fukui with concerns of severe pain associated with a firm mass in the anus. Her medical history included a hysterectomy for cervical cancer at the age of 36 years. She was a non-smoker and did not drink alcohol. The findings of a physical examination revealed no abdominal masses or tenderness; however, the rectal examination revealed a firm nodular mass at the anal verge advancing along the left side of the rectum. Endoscopic evaluation revealed an anorectal mass (Fig. 1a), and biopsy results were consistent with moderately differentiated squamous cell carcinoma. The laboratory tests showed elevated levels of squamous carcinoma antigen at 3.9 µg/L (normal < 1.5 µg/L) and normal levels of serum carcinoembryonic antigen and carbohydrate antigen 19-9. Enhanced computed tomography (CT) revealed thickening in the left lateral wall of the anal canal (Fig. 1b). CT and small paramagnetic iron oxide magnetic resonance imaging showed several vague low-density foci in both hepatic lobes, the largest measuring 2.5 cm, that were suspicious of metastatic disease (Fig. 2a, b). The cancer was classified as stage IV, T4N0M1.

**Fig. 3:**
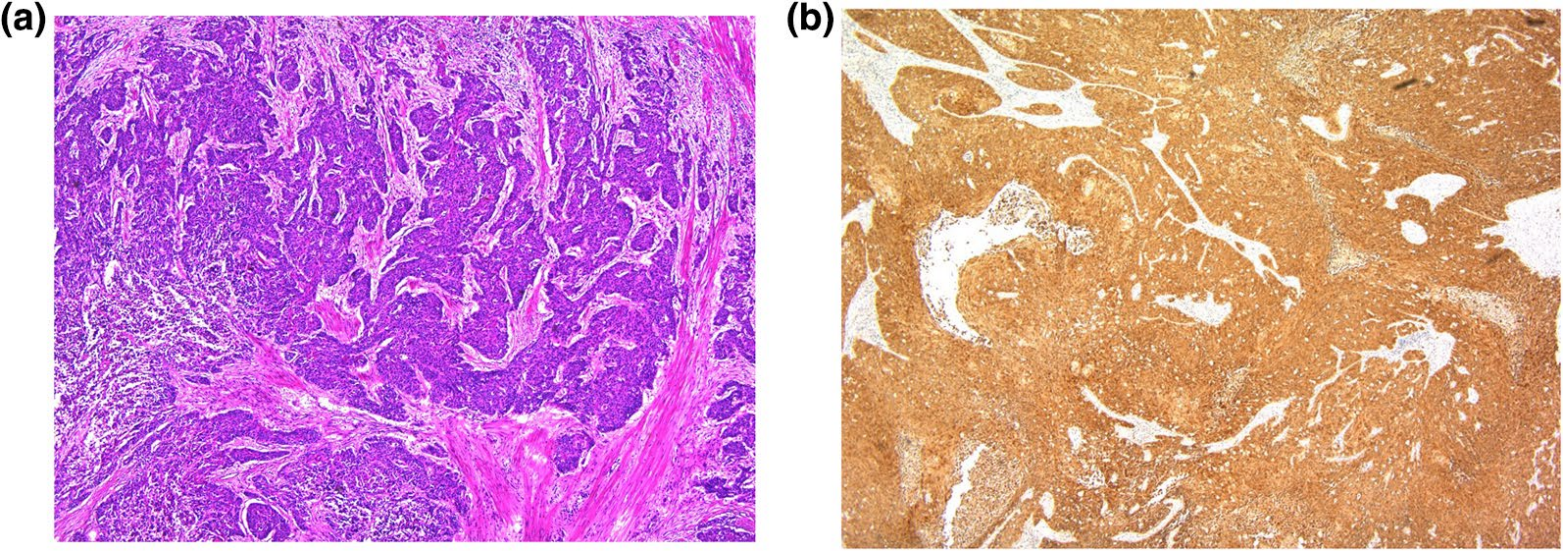
**a** Histopathological examination of the resected specimen showed moderately differentiated squamous cell carcinoma (hematoxylin–eosin stain, original magnification ×40). **b** Immunohistochemistry for p16 showing strong immunoreactivity (original magnification ×40)

The patient underwent rectal amputation with lateral lymph node dissection (D3). Macroscopically, a type 2 tumor was found half way around the wall of the anal canal. The tumor was histopathologically diagnosed as moderately differentiated squamous cell carcinoma (Fig. 3a). No metastasis in the regional or lateral lymph nodes was observed. Additionally, immunohistochemical staining revealed that the tumor was positive for p16 (Fig. 3b), which indicates the presence of human papilloma virus (HPV).

**Fig. 4:**
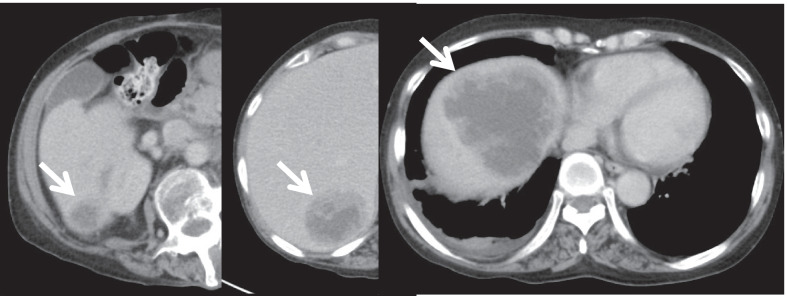
Abdominal CT scan at 3 months postoperatively showing marked increase in multiple liver metastases (white arrow). *CT* computed tomography

Ileus was observed on the 5th postoperative day. Thereafter, a small bowel decompression tube was inserted, and a conservative treatment was performed. On the 14th postoperative day, the patient underwent an ileus release procedure. The cause of the ileus was adhesion of the small intestine to the pelvic floor. Postoperatively, we found a recurrence of ileus and a pelvic abscess; therefore, 1 month later, we performed an ileus release again and abscess drainage. The patient had severe adhesions in the abdominal cavity, which made dissection of the intestine and drainage of the abscess difficult. Postoperatively, the midline wound was contiguous with a vulnerable area of the small intestine, and a small bowel leak was formed. Healing was achieved by continuous aspiration of the small bowel leak. It took approximately 3 months from the initial surgery to the healing of the complication. The abdominal CT scan after treatment for complications showed notable enlargement of the liver metastases (Fig. 4).

**Fig. 5:**
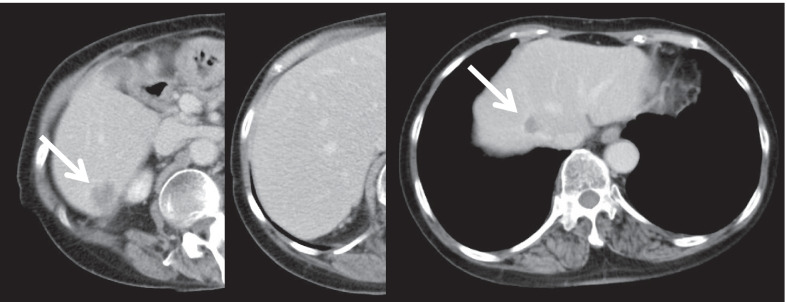
Abdominal CT scan showing reduction of liver metastases by chemotherapy (white arrow). *CT* computed tomography

Because the patient was physically exhausted due to the complications, she underwent RF hyperthermia as first-line treatment for the liver metastases, as this modality is less burdensome. We combined anticancer drug treatment with mitomycin c (MMC) and cisplatin, which is sensitized by RF hyperthermia. RF hyperthermia was administered once a week for 5 weeks. As the next line of treatment, systemic chemotherapy with 5-fluorouracil (5-FU) and cisplatin (CDDP) was administered, which is recommended for stage IV anal canal squamous cell carcinoma in the National Comprehensive Cancer Network (NCCN) guidelines [6]. To meet the patient’s request for outpatient chemotherapy, low-dose CDDP (10 mg) and 5-FU (600 mg/m^2^) were administered once a week as an outpatient. She underwent this treatment regimen until 22 months postoperatively and showed notable reduction in the size of the liver metastases (Fig. 5). The patient experienced an allergic reaction to CDDP, and the regimen was changed to a combination of TS-1 and docetaxel, which was continued until 58 months postoperatively.

**Fig. 6:**
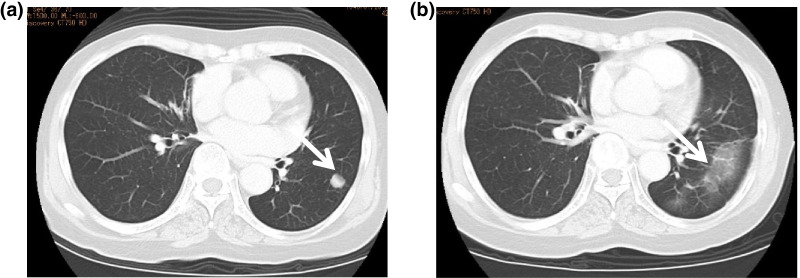
**a** Chest CT scan showing the presence of lung metastases (white arrow). CT, computed tomography. **b** Chest CT scan showing the disappearance of lung metastases after radiotherapy (white arrow). *CT* computed tomography

**Fig. 7:**
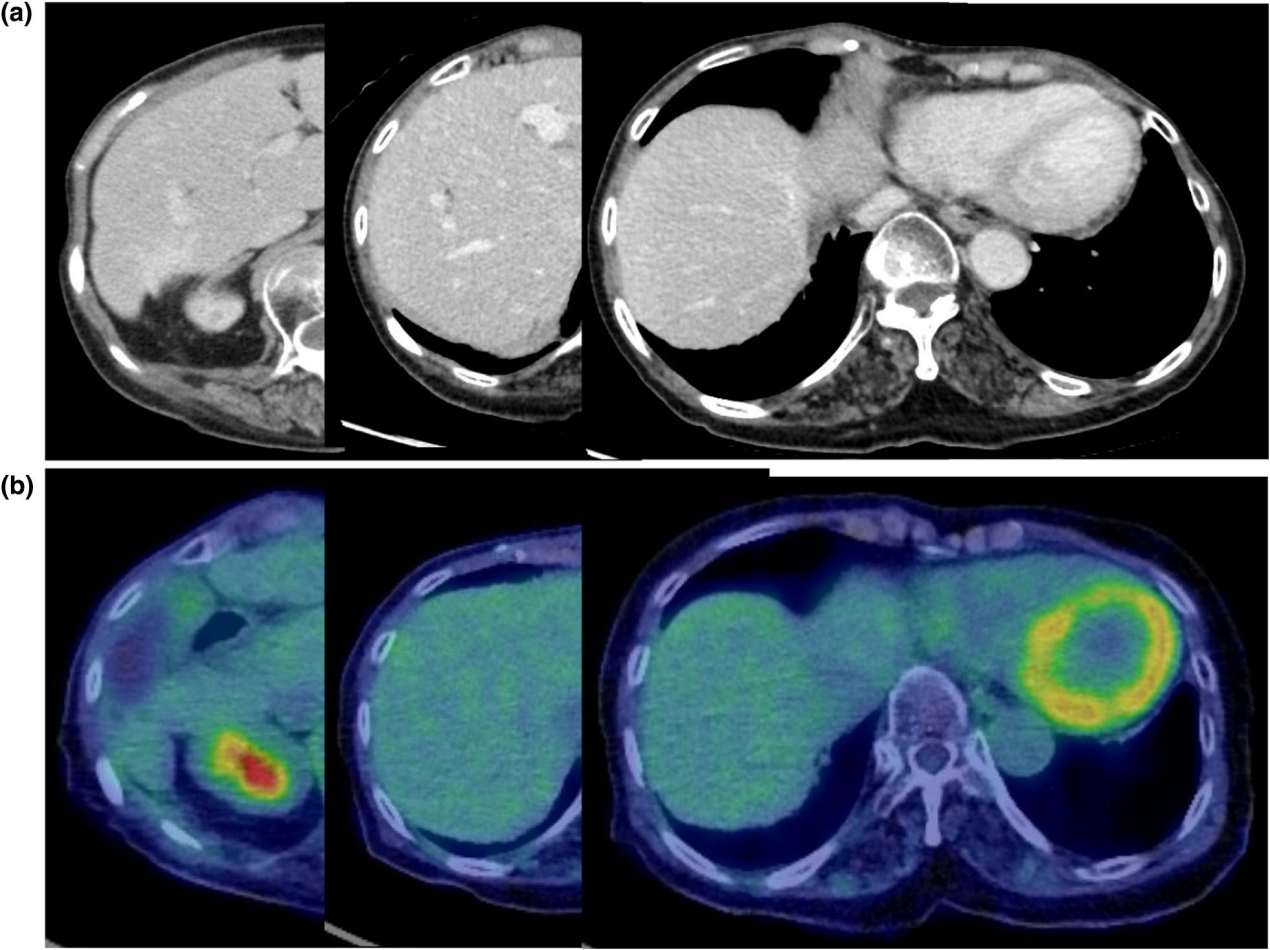
**a** Abdominal CT showing complete remission of liver metastases. *CT* computed tomography. **b** PET–CT scan showing no sign of recurrence. *PET–CT* positron emission tomography–computed tomography

During the course of systemic chemotherapy at 16 months postoperatively, one metastasis appeared in the left lower lung field (Fig. 6a) and radiation therapy was performed. After receiving a total dose of 50 Gy, the lung metastases disappeared and remained in complete remission (CR) for over 9 years (Fig. 6b). For the remnant liver metastases at segment 6 and 8, 50 Gy radiotherapy was administered at 23 and 49 months postoperatively. The liver metastases resolved 54 months postoperatively (Fig. 7a, b), and the anticancer drug treatment was discontinued. The patient remained in CR for 10 years after the initial presentation.

## Discussion

Anal cancer is an uncommon malignancy accounting for 2% of all gastrointestinal malignancies, with a reported incidence of 7000 cases in the United States in 2014 [5, 7]. Specifically, squamous cell carcinoma accounts for 75% of anal cancer cases in the United States and Europe [4, 8]. Despite its rarity, the incidence of anal cancer has been increasing over the past three decades [9] likely due to changing trends in sexual behavior, smoking, HPV or HIV infection, and immune suppression in transplant recipients [10].

Large randomized trials have shown that chemoradiation provides better locoregional control and colostomy-free survival than surgical treatment for locally advanced tumors or carcinomas with lymph node metastasis [2, 3, 11]. The NCCN guidelines recommend 5-FU + MMC chemotherapy and radiotherapy for patients with only a primary tumor [6] and long-term survival and curative cases have been reported [12, 13].

In anal squamous cell carcinoma, distant metastasis has been observed in 5% to 8% of cases at initial presentation and 10% to 20% of cases after curative local treatment [1, 6, 14]. The prognosis of patients with metastatic disease remains poor, with an estimated 5-year OS rate of only 13% [15] and a median OS of 8–12 months [16–19]. Due to the rarity of metastatic anal squamous cell carcinoma, evidence-based guidelines defining the optimal treatment are lacking. Nevertheless, for metastatic anal squamous cell carcinoma, the NCCN guidelines recommend 5-FU + CDDP chemotherapy, which has been recommended for many years based on small cohort studies [14, 20]. However, response rates to this regimen are low, and complete responses are rare. Reports have suggested the efficacy of docetaxel [21], paclitaxel [22], doxorubicin, and cetuximab [23–25], but no second-line therapy has been established.

To treat distant metastasis, local radiotherapy is indicated in addition to systemic chemotherapy when local tumors are observed [6]. Hoyer et al. reported on the effectiveness of radiotherapy for liver metastases in various cancers [26]. They found that the median OS after radiotherapy ranges from 10 to 34 months and the 2-year OS rate ranges from 30 to 83%, with some long-term survivors. They further reported mild-to-moderate side effects. They report states that radiotherapy is more effective in cases of liver metastases related to diseases other than colorectal cancer than in cases of colorectal cancer-related liver metastases and is ideal for three or fewer metastases with size of 6 cm or less. Radiotherapy is also effective for lung metastases of colorectal cancer [27] and cervical cancer [28], and may be effective for lung metastases of anal squamous cell carcinoma.

Although no studies have reported evidence of the efficacy of resection of distant metastasis alone, some have reported improved prognosis from radiation in combination with resection of distant metastasis [9]. Pawlick et al. reported on 27 patients in a multicenter analysis of hepatic resection for anal canal squamous cell carcinoma metastasized to the liver. Outcomes for this group showed encouraging results, with a disease-free survival (DFS) of 9.6 months and a 5-year OS rate of 23% [29]. They also proposed that poor prognostic factors in liver resection cases include liver metastases larger than 5 cm and positive resection margins.

Multidisciplinary treatment (systemic chemotherapy along with surgery and/or CRT, CRT alone, or radiofrequency ablation) for anal squamous cell carcinoma patients with distant metastasis is effective and results in long-term survival. For example, Eng et al. reported good results in 33 patients who underwent multidisciplinary treatment, with a median progression-free survival (PFS) of 16 months and DFS of 52 months, and a clear advantage of multidisciplinary treatment was identified in comparison to patients treated with systemic chemotherapy who experienced a median PFS and OS of 5 and 17 months, respectively [30]. In addition, six cases of patients undergoing multidisciplinary treatment resulting in a PFS of more than 4 years have been reported [5, 20, 31, 32]. ESMO–ESSO–ESTRO guidelines recognize the potential of multidisciplinary treatment and comment that those with small-volume or oligometastatic disease should be evaluated in a multidisciplinary fashion to determine surgery or CRT options [1].

Eng et al. reported that systemic chemotherapy with 5-FU plus cisplatin or carboplatin plus paclitaxel is effective, and advocated considering local treatment such as surgery, stereotactic radiotherapy, or radiofrequency ablation if the patient responds to these systemic chemotherapy treatments. It was further suggested that a multidisciplinary team should be involved in deciding on the local treatment strategy [30]. Gnanajothy et al. indicated the sequence of multidisciplinary treatment as systemic chemotherapy, surgery, and radiation chemotherapy. In the reported case, liver metastases were resected after systemic chemotherapy, followed by radiation chemotherapy for the primary tumor, with good results [5]. Lupinacci et al. performed systemic chemotherapy followed by local treatment of the primary tumor, followed by resection of liver metastases. They stated that although the ideal treatment sequence is under discussion, it is important to determine the patients who would benefit from a more aggressive treatment strategy that addresses both local disease and solitary metastases [33].

In Japan, squamous cell carcinoma of the anal canal accounts for only 24% of all anal canal cancers, and surgical treatment was performed initially in the present case because an adequate treatment plan had not been established previously and the patient was under severe pain. Yamada et al. reported changes in the percentage of patients treated with CRT for anal squamous cell carcinoma in Japan. According to the report, surgery was performed in 14.3% of the cases from 1991 to 2000, 62.2% of the cases from 2001 to 2010; and 84.3% of the cases from 2011 to 2015 [34].

In our patient, a marked increase in liver metastases was observed after the surgery, which was considered to be a prognostic factor. RF hyperthermia was administered as a local treatment. After these treatments enabled local control, systemic chemotherapy was administered to treat the local residual tumor and prevent distant metastasis. During the course of the treatment, radiotherapy was performed for pulmonary metastasis with marked efficacy. Liver metastases did not resolve after systemic chemotherapy; however, since radiotherapy for lung metastases was effective, it was also administered to the liver metastases, which proved effective. In this case, complications such as postoperative ileus and intra-abdominal abscess were observed, and since a high degree of adhesion was suspected, laparotomy was difficult. Our patient demonstrated CR at 54 months after surgery without recurrence for 10 years, which is considered extremely rare. However, there are few reports of CR and long-term prognosis in patients with metastatic squamous cell carcinoma of the anus [20, 31, 32], and very few cases of CR without hepatic resection of liver metastases [35–37]. These cases are similar to ours, with the combined treatment approach resulting in improved progression-free survival without hepatic resection of liver metastases as summarized in Table 1.

**Table 1:** Previously published cases of distant metastases with complete response without resection of liver metastases

Author	OS (years)	Distant metastases	Treatment approach
Joe et al. [35]	4	Liver, bone	MMC + capecitabine + Ra
Haydon [36]	7	Liver, lung	CDDP + 5-Fu
Maulik et al. [37]	10	Liver	CDDP + 5-Fu → MMC + 5-Fu + Ra

*5-FU* 5-fluorouracil, *CDDP* cisplatin, *MMC* mitomycin C, *OS* overall survival, *Ra* radiation therapy

A commonly used regimen is 5-FU administered as a continuous infusion over 5 days at a dose of 1000 mg/m^2^/day with 100 mg/m^2^ of CDDP on day 2; the cycle is repeated every 4 weeks. In this case, the patient requested outpatient treatment and was administered a low-dose of 5-FU and CDDP therapy. It is believed that CDDP has the ability to enhance the cell killing effect of 5-FU even when administered in small doses [38], as observed in this case. However, our patient experienced an allergic reaction to CDDP, and chemotherapy with TS-1 and docetaxel was effectively administered according to the standard treatment protocol for esophageal squamous cell carcinoma in Japan.

Furthermore, Rogers and Eng reported that taxanes are active components, with or without combination antineoplastics, in patients with metastatic anal squamous cell carcinoma [14].

Clinical trials are being conducted by the Japan Clinical Oncology Group to investigate the efficacy of preoperative radio chemotherapy with TS-1 and MMC. If these treatments are proved effective, TS-1 will become a new key drug for anal squamous cell carcinoma.

Anal squamous cell carcinoma has been linked to HIV and other immunodeficiencies. Nivolumab activates the T cell immune response by inhibiting the binding of PD-1 and PDL-1 and is effective against anal squamous cell carcinoma [35, 39].

Moreover, cetuximab may be an effective treatment for anal squamous cell carcinoma because EGFR is overexpressed in many cases, while KRAS, NRAS, and BRAF mutations are not observed in most cases [14, 40–42]. Rogers et al. reported that EGFR antibodies were used in second- and third-line treatments and disease control was possible for 59% of the patients [43]. In addition, Kim et al. reported that 46% of the patients had well-controlled disease, with a PFS of 4.4 months and an OS of 11.4 months [44].

As reported, various drugs may be effective in treating anal squamous cell carcinoma, and multidisciplinary treatment may aid in disease control for metastatic cases. Randomized trials are needed to determine which treatments are effective in treating metastatic anal squamous cell carcinoma.

## Conclusion

Herein, we reported a rare case of long-term CR in a patient with metastatic anal squamous cell carcinoma. Multidisciplinary treatment may be effective for such patients. Furthermore, the addition of a new anticancer therapy may improve the overall prognosis of squamous cell carcinoma.

## Data Availability

The datasets during and/or analyzed during the current study are available from the corresponding author on reasonable request.

## Abbreviations

RF hyperthermia: Radiofrequency hyperthermia
CRT: Combined chemoradiotherapy
CT: Computed tomography
HPV: Human papilloma virus
MMC: Mitomycin C
5-FU: 5-Fluorouracil
CDDP: Cisplatin
NCCN: The National Comprehensive Cancer Network
CR: Complete remission
HIV: Human immunodeficiency virus
OS: Overall survival
DFS: Disease-free survival
PFS: Progression-free survival
Ra: Radiation therapy
